# The role of interaction between vitamin D and VDR FokI gene polymorphism (rs2228570) in sleep quality of adults

**DOI:** 10.1038/s41598-024-58561-2

**Published:** 2024-04-07

**Authors:** Luiz Antônio Alves de Menezes-Júnior, Thais da Silva Sabião, Samara Silva de Moura, Aline Priscila Batista, Mariana Carvalho de Menezes, Júlia Cristina Cardoso Carraro, George Luiz Lins Machado-Coelho, Adriana Lúcia Meireles

**Affiliations:** 1 Postgraduate Program in Health and Nutrition, School of Nutrition, Federal University of Ouro Preto, R. Diogo de Vasconcelos, 122, Ouro Preto, MG Brazil; 2 Research and Study Group On Nutrition and Public Health (GPENSC), Federal University of Ouro Preto, Ouro Preto, Brazil; 3 Postgraduate Programs in Biological Sciences, Institute of Biological Sciences, Federal University of Ouro Preto, Ouro Preto, MG Brazil; 4 School of Medicine, Federal University of Ouro Preto, Ouro Preto, MG Brazil; 5 Department of Clinical and Social Nutrition, School of Nutrition, Federal University of Ouro Preto, Ouro Preto, MG Brazil

**Keywords:** Single-nucleotide polymorphism, Vitamin D receptor, Nutrigenetics, Population genetics, Sleep disorders, Nutrition, Epidemiology

## Abstract

To evaluate association of vitamin D with sleep quality in adults and the influence of *VDR*-gene polymorphism FokI (rs2228570;A > G). Cross-sectional population-based study in adults, conducted in Brazil. The outcome was sleep-quality, evaluated by the Pittsburgh Sleep Quality Index. Vitamin D was determined by indirect electrochemiluminescence and classified as deficiency (VDD), 25(OH)D < 20 ng/mL in a healthy population or 25(OH)D < 30 ng/mL for groups at risk for VDD. FokI polymorphism in the *VDR*-gene was genotyped by qPCR and classified as homozygous wild (FF or AA), heterozygous (Ff or AG), or homozygous mutant (ff or GG). Multivariate logistic analysis was used to estimate the association between vitamin D and FokI polymorphism with sleep-quality. In a total of 1674 individuals evaluated, 53.6% had poor-sleep-quality, 31.5% had VDD, and the genotype frequency of the FokI polymorphism was 9.9% FF, 44.6% Ff, and 45.5% ff. In multivariate analysis, individuals with VDD had 1.51 times the chance of poor-sleep-quality, and individuals with the ff genotype had 1.49 times the chance of poor-sleep-quality (OR:1.49;95%CI:1.05–2.12) when compared to individuals with the FF or Ff genotype. In the combined analysis, individuals with VDD and ff genotype had more chance of poor-sleep-quality than individuals with sufficient vitamin D and genotype Ff or FF (OR:2.19;95%CI:1.27–3.76). Our data suggest that VDD and VDR FokI gene polymorphism are associated with poor-sleep-quality, and combining the two factors increases the chance of poor-sleep-quality compared to separate groups.

## Introduction

Vitamin D performs several functions, primarily by regulating osteomineral physiology, particularly calcium metabolism^1^. However, current evidence indicates that the pleiotropic effects of vitamin D and its metabolites extend beyond bone mineral metabolism and parathyroid gland activity, with products linked to other potential areas that are mainly sleep-related^2,3^. The precise causal mechanisms underlying this association remain elusive, but hypotheses have been proposed regarding the involvement of vitamin D receptors located intracellularly in areas of the brain responsible for regulating the sleep–wake cycle as well as the role of pro-inflammatory mediators^4,5^. Experimental evidence suggests an inverse relationship between sleep regulatory substance and vitamin D levels^4^. However, the biological effects of vitamin D rely on its binding to the vitamin D receptor (VDR), which interacts with other coregulatory proteins to modulate the transcription of target genes responsive to vitamin D. Consequently, variations in the VDR gene can influence the binding affinity of the receptor for vitamin D, thereby altering the response to this vitamin across different tissues^6^.

The FokI polymorphism (rs2228570) within the first exon has been extensively studied and characterized. This polymorphism results in a protein with reduced transcriptional activity and a diminished binding affinity for vitamin D^7,8^. Such alterations can affect the physiological responses to vitamin D in tissues, potentially affecting those associated with sleep regulation. Hence, it is plausible to hypothesize that this polymorphism in VDR may yield functional consequences similar to those observed in vitamin D deficiency, suggesting a potential link to poor sleep quality. Additionally, evidence suggests that this polymorphism may be associated with health outcomes that are intrinsically linked to sleep, such as major depressive disorder^9^, cognitive functioning^10^, and fibromyalgia^11^, with sleep possibly acting as a mediator of these relationships. Moreover, initial investigations have suggested a potential association between the FokI polymorphism and obstructive sleep apnea, a prominent sleep disorder^12,13^.

Therefore, we hypothesized that vitamin D deficiency and the presence of a homozygous polymorphic (ff or GG) genotype are positively associated with poor sleep quality. Therefore, this study aimed to evaluate the association between vitamin D deficiency, the *VDR* gene polymorphism FokI (rs2228570), and sleep quality.

## Methods

### Study design

This is a cross-sectional, population-based survey by multistage probability cluster sampling, conducted between October and December 2020 in two medium-sized cities (Ouro Preto and Mariana) in the south-central region of Minas Gerais, known as Iron Quadrangle, one of the largest iron ore producing areas in Brazil.

The data collection was carried out in the sample design in three steps: census sector, household, and resident, with the representativeness of the different socioeconomic strata (< 1 minimum wage, 1 to 3 minimum wages, and ≥ 4 minimum wages) guaranteed in the final sample.

The sample size was calculated using the OpenEpi tool (https://www.openepi.com/Menu/OE_Menu.htm), considering the prevalence of poor sleep quality considered as 50%, according to previous epidemiological surveys in adults, confidence level of 95%, design effect equal to 1.5 and precision of 5%. To ensure accuracy, the sample was increased by 20% to compensate for eventual losses due to refusals, the absence of the resident selected for the study, and closed households. Based on the sample calculation, 687 and 684 individuals should have been interviewed in Ouro Preto and Mariana, totaling 1371 individuals in the two cities evaluated. During the data collection process, we evaluated 1762 individuals, of which 88 were not analyzed due to insufficient blood samples for dosing of 25-hydroxyvitamin D and extraction of viable DNA for genotyping. Therefore, 1674 individuals representing adult residents in the urban areas of the two cities were included in this study. Face-to-face interviews were conducted in the resident’s homes using an electronic form by the interviewer. The questionnaire included sociodemographic and economic aspects, living habits, general health conditions, and sleep quality. All procedures were performed according to the Brazilian guidelines and standards for research involving human beings of the Declaration of Helsinki and were approved by the Research Ethics Committee (Ethics Submission Certificate no. 32815620.0.1001.5149). This study followed reported guidelines dictated by the Strengthening the Reporting of Observational Studies in Epidemiology (STROBE). For more details on data collection, see Meireles et al.^14^.

### Sleep quality

Sleep quality was evaluated by the Pittsburgh Sleep Quality Index (PSQI) (Buysse et al., 1989). The Brazilian version of the PSQI had an overall reliability coefficient (Cronbach’s α) of 0.82, indicating a high degree of internal consistency^15^. This instrument is composed of 19 questions categorized into seven components, each component scoring from 0 to 3: subjective sleep quality (C1), sleep latency (C2), sleep duration (C3), habitual sleep efficiency (C4), sleep disturbances (C5), sleep medication use (C6), and daytime dysfunction (C7). The sum of the scores produces an overall score ranging from 0 to 21, where the highest score indicates poorer sleep quality^16^. In this study, sleep quality was classified as “good sleep quality” when the PSQI score was less or equal to 5 and as “poor sleep quality” when the PSQI was greater than 5^15,16^.

### Biological sample collection

Blood collection was performed by a trained professional by puncture in the region of the cubital fossa. For collection, a 7.5 mL S-Monovette^®^ (Sarstedt) serum gel tube was used for vitamin D analysis; and a 2.7 mL S-Monovette^®^ (Sarstedt) collection tube containing sodium fluoride/EDTA to obtain whole blood for molecular biology analyses. Subsequently, the samples were verified and transported to the Laboratory of Epidemiology (LEPI) of the School of Medicine at the Federal University of Ouro Preto (UFOP). In the laboratory, whole blood was stored in a freezer at − 20 °C, and the serum tubes were centrifuged at 2500 rpm for 15 min, and stored at − 80 °C for posterior analysis.

### Vitamin D serum concentrations

Vitamin D was determined by indirect electrochemiluminescence with competition principle in the Access 2 Immunoassay System^®^ (Beckman Coulter, USA) with a Roche Diagnostics^®^ commercial kit (Roche, Switzerland). For intra-laboratory analysis, the coefficient of variation of the method ranges from 6.1 to 7.5%, and the correlation coefficient with LC–MS/M was 0.92 (data provided by the manufacturer). Vitamin D concentrations were classified as a deficiency when 25(OH)D < 20 ng/mL in a healthy population and 25(OH)D < 30 ng/mL for groups at risk for vitamin D deficiency (body mass index ≥ 30 kg/m^2^, age ≥ 60 years, individuals with brown or black skin color, pregnant women, and presence of cancer, diabetes or chronic kidney diseases)^17^.

### VDR gene FokI polymorphism

The genomic DNA extraction was performed with Wizard^®^ Genomic DNA Purification kit (Promega, USA) according to the manufacturer’s protocol. After extraction, the DNA was maintained for 24 h in a hydration solution and dosed by fluorimetry (Qubit 2.0 Fluorometer, Invitrogen^®^). The DNA samples were stored at -20 °C until use.

In this study, we evaluated the allelic discrimination of the FokI polymorphism (rs2228570 A > G) in the *VDR* gene, which consists of a nucleotide base change from adenosine (A) to guanine (G). The analysis was performed via the real-time PCR (qPCR) technique using the TaqMan^®^ SNP Genotyping Assay System (Applied Biosystems, Foster City, USA) consisting of fluorescently labeled (FAM and VIC) probes (Applied Biosystems, Foster City, CA) in the 7500 Fast Real-Time PCR Systems equipment (Applied Biosystems, USA) (initial denaturation: 10 min, 95 ºC; ringing: 15 s, 40 cycles of 95 ºC; and final extension: 1 min, 60 ºC). Specific primers from Thermo Scientific (Assay ID__12060045_20) were used according to the manufacturer’s instructions.

### Covariates

The questionnaire included variables for possible confounding controls in the association between vitamin D and sleep quality analysis^5,18,19^. The sociodemographic variables evaluated were sex (female or male), age group (18–34 years; 35–59 years; ≥ 60 years), marital status (single or married), current family income (≤ 2 minimum wages; > 2 to ≤ 4 minimum wages; > 4 minimum wages), and education level (< 8 years; 9–11 years; ≥ 12 years of study). Self-reported race/skin color was evaluated using the categories proposed by the Brazilian Institute of Geography and Statistics (IBGE) and were categorized into white, black, brown, and other races/skin colors (indigenous and yellows).

Health conditions evaluated were self-reported chronic diseases (hypertension, diabetes, asthma, lung disease, chronic kidney disease, cancer, heart or thyroid disease), which were dichotomized into morbidity (reporting at least one condition) and without morbidity (no disease). Furthermore, the following lifestyle variables were assessed: current smoking (yes or no), current alcohol drinking (yes or no), and physical activity (physically active when they reached at least 150 min of moderate-intensity aerobic physical activity or at least 75 min of vigorous-intensity aerobic physical activity per week, or physically inactive when the recommendations were not reached) (WHO, 2020). Nutritional status was evaluated by body mass index (BMI) from self-reported height (cm) and weight (kg). BMI was classified as underweight (BMI < 18.5 kg/m^2^ if aged < 60 years; BMI < 23.0 kg/m^2^ if old ≥ 60 years), eutrophic (BMI 18.5–24.9 kg/m^2^ if aged < 60 years; BMI 23.0–28.0 kg/m^2^ if aged ≥ 60 years), overweight (BMI 25.0–29.9 kg/m^2^ if old < 60 years; BMI 28.0–29.9 kg/m^2^ if aged ≥ 60 years), and obesity (BMI ≥ 30.0 kg/m^2^) according to WHO and PAHO for adults and elderly, respectively^20,21^

The anxiety and depression symptoms were evaluated by the Generalized Anxiety Disorder scale (GAD-7) and Patient Health Questionnaire (PHQ-9) scale, respectively^22,23^.For both scales, scores equal to or above 10 were considered to determine the presence of anxiety and depression symptoms^22,23^.

Daily sunlight was assessed quantitatively from the following questions: “From Monday to Sunday, how many times a week, and for how long are you exposed to the sun?” Subsequently, the average daily sunlight was calculated from the following formula: weekly frequency of sunlight (0–7 days) x daily time of sunlight (minutes)/7]. Moreover, we evaluated whether individuals used any vitamin D dietary supplements by self-reporting, “In the past three months, have you used a vitamin-based dietary supplement, such as vitamin D or cholecalciferol or cod oil supplementation?” (yes or no).

### Statistical analysis

For statistical analyses, the Stata/MP program (version 15.0) was used with an alpha of 5%. All analyses were performed considering the study design and sampling weighting factors using the “svy” package. To characterize the sample, the distribution of continuous variables was assessed using the Shapiro–Wilk test, and all followed a parametric distribution. Subsequently, continuous variables were expressed as means and 95% confidence intervals (CI), and categorical variables were described as relative frequencies and 95% CI.

Allele frequencies were estimated using the gene counting method. Departure from Hardy–Weinberg equilibrium (HWE) was assessed via an exact two-sided probability test using the formula provided by Weir (1996).

**Figure 1:**
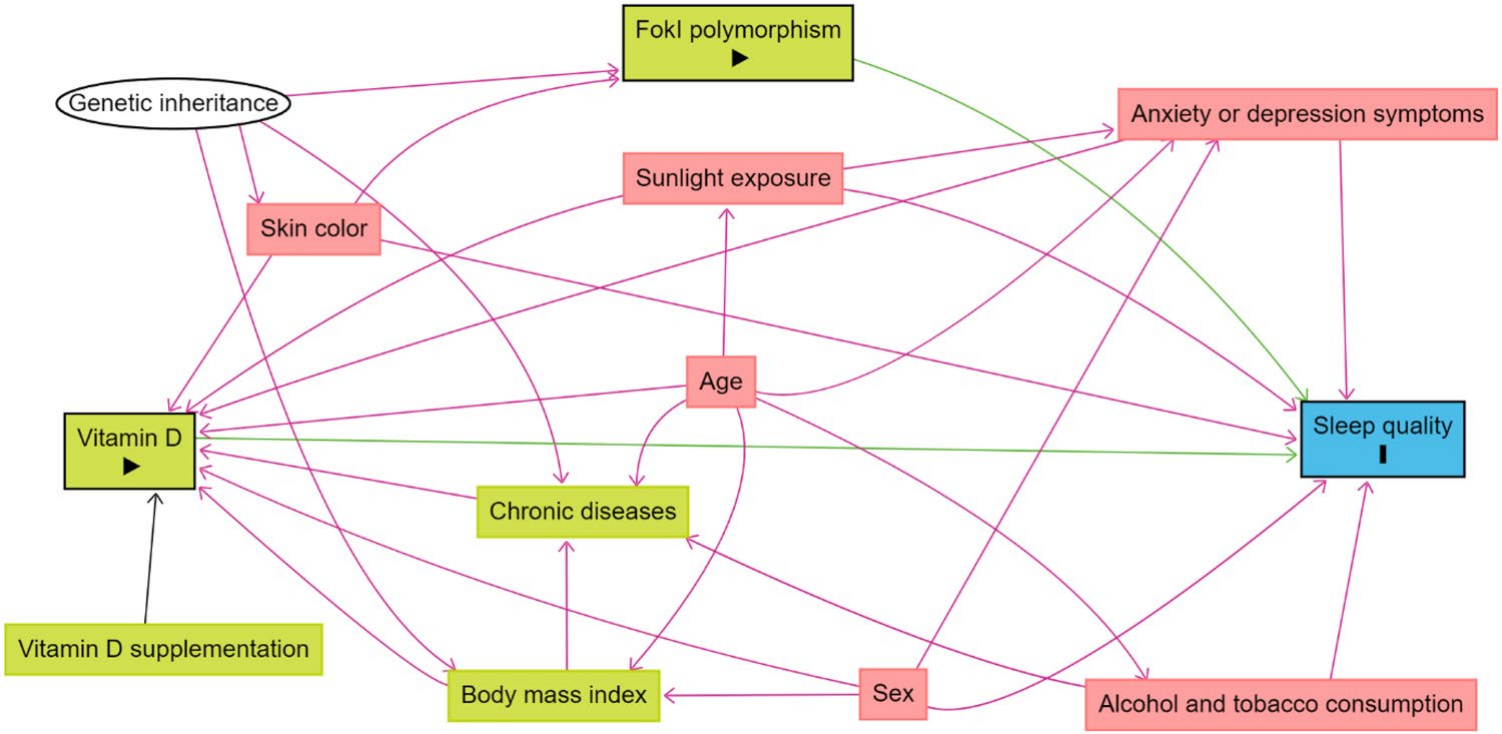
Directed acyclic graph (DAG) on vitamin D and VDR FokI genotype with sleep quality in adults. The variable in green and with the “►” symbol inside the rectangle was the exposure variable; those in blue and with the “**❙**” symbol inside the rectangle was the outcome variable; variables represented in white circles represent variables not directly measured in the study; variables in green are the antecedents of the exposure variable; and those in red are antecedents of the outcome and exposure variables.

A theoretical causality model based on a directed acyclic graph (DAG) was developed according to the exposure variables (vitamin D status and FokI polymorphism), outcome (sleep quality), and covariates using the online software Dagitty version 3.2. The causal connections, represented by arrows, were established between the variables (Fig. 1). Supplementary Table S1, “Health-related pathways overview illustrated in the directed acyclic graph (DAG),” provides a visual representation of the theoretical model developed for this study. To avoid unnecessary adjustments, spurious associations, and estimation errors, a backdoor criterion was used to select the minimum set of confounding variables required to fit the analyses. The model was adjusted using the following minimum and sufficient set of variables: age, sex, skin color, alcohol and tobacco consumption, body mass index, anxiety and depression symptoms, and exposure to sunlight.

Therefore, vitamin D deficiency and FokI polymorphisms were evaluated independently and tested for their combined relationship with sleep quality. A combined interaction analysis was also performed. The variance inflation factor assessed collinearity between covariates with the “subsetByVIF” package considering a maximum cutoff point of 10 (VIF < 10).

### Ethical considerations

All procedures followed the Brazilian standards for research involving human beings, the Declaration of Helsinki. This study was approved by the Research Ethics Committee of the Federal University of Minas Gerais (Ethics Submission Certificate No. 32815620.0.1001.5149) on September 22, 2020 (approval number: 4292475). Written informed consent was obtained from all participants.

## Results

### Characteristics of study participants

Table 1 shows the sociodemographic characteristics and health status of the study participants. Of the participants, 51.7% were female; the most prevalent age group was 35–59 years (45.4%); most were black, brown, or other skin colors (74.4%); married (53.5%); had 9–11 years of education (39.9%); and had a family income equal to or less than two minimum wages (45.1%). Supplementary Table S2 provides further insights into the sociodemographic and health conditions according to vitamin D levels, while Supplementary Table S3 presents similar information categorized by the FokI polymorphism.

**Table 1:** Sociodemographic and health conditions in adults during the COVID-19 pandemic, COVID-Inconfidentes Study (2020).

Characteristics	Prevalence	Lower 95% confidence bound	Upper 95% confidence bound
Sociodemographic			
Sex			
Male	48.3	41.1	55.6
Female	51.7	44.4	58.9
Age			
Years, mean (95%CI)	43.4	42.0	44.8
18 to 34 years	36.7	32.3	41.3
35 to 59 years	45.4	41.0	49.9
≥ 60 years	17.9	14.5	21.7
Skin color^a^			
White	25.6	20.8	31.2
Black, brown and others	74.4	68.6	79.4
Marital status^b^			
Married	53.5	47.1	59.8
Not married	46.5	40.2	52.8
Education			
0 to 8 years	30.2	25.4	35.5
9 to 11 years	39.9	35.4	44.5
≥ 12 years	29.9	24.4	36.1
Family Income^c^			
≤ 2 MW	45.1	40.2	50.1
> 2 to ≤ 4 MW	30.1	25.5	35.2
> 4 MW	24.8	20.0	30.3

Health conditions			
Vitamin D^d^			
Mean, ng/dL	26.2	25.2	27.1
Sufficiency	68.5	62.3	74.0
Deficiency	31.5	26.0	37.7
FokI polymorphism^e^			
FF or Ff	54.5	48.4	60.5
ff	45.5	39.5	51.6
Sleep quality^f^			
Good	46.4	42.2	50.7
Poor	53.6	49.3	57.8
Chronic diseases^g^			
No	60.6	55.0	65.9
Yes	39.4	34.0	45.0
Smoking			
No	83.0	78.4	86.8
Yes	17.0	13.2	21.6
Alcohol consumption			
No	42.3	36.0	48.8
Yes	57.7	51.2	63.9
Body mass index (BMI)^h^			
BMI, kg/m^2^	26.6	26.1	27.0
Eutrophic	42.0	35.3	48.9
Underweight	2.6	1.8	3.8
Overweight	36.8	29.3	45.1
Obesity	18.6	15.2	22.5
Mental health^i^			
Presence of anxiety symptoms	23.4	19.5	27.7
Presence of depression symptoms	15.8	12.7	19.4
Exposure to sunlight^j^			
Daily sunlight (hours/day)	3.51	2.47	4.56
Vitamin D supplementation			
No	93.5	91.5	95.1
Yes	6.5	4.9	8.5

^a^The participants were categorized into those with white or black, brown, and others race/skin colors (indigenous and yellows).

^b^Not married: Widowed, divorced, single.

^c^Minimum wage value: BRL 1,045.00 ≈ USD 194.25 (1 USD = 5.3797 BRL).

^d^Vitamin D deficiency: 25(OH)D < 20 ng/mL for a healthy population and < 30 ng/mL for groups at risk for vitamin D deficiency.

^e^Genotype frequency (FokI): FF or AA- homozygous wild, Ff or AG- heterozygous and ff or GG- homozygous mutant.

^f^Poor sleep quality determined by PSQI ≥ 5.

^g^Chronic disease: medical diagnosis of at least one chronic disease.

^h^Underweight (BMI < 18.5 kg/m^2^ if < 60 years or BMI < 22.0 kg/m^2^ if > 60 years), eutrophic (BMI 18.5–24.9 kg/m^2^ if < 60 years or BMI 22.0–27.9 kg/m^2^ if > 60 years), overweight (BMI 25.0–29.9 kg/m^2^ if < 60 years or BMI 28.0–29.9 kg/m^2^ if > 60 years), obese (BMI > 30.0 kg/m^2^).

^i^The GAD-7 and PHQ-9 scales were used to determining the presence of anxiety and depression symptoms, respectively.

^j^Daily sunlight was calculated from the following formula: [weekly frequency of sunlight (0 – 7 days) x daily time of sunlight (minutes)/7]).

Concerning health conditions, 31.5% of the individuals had vitamin D deficiency. The mean daily sunlight was 3.51 h; 6.5% used any vitamin D supplementation; 53.3% had poor sleep quality; 39.4% had chronic diseases; 57.7% and 17.0% consumed alcohol or tobacco, respectively; 36.8% and 18.6% were overweight or obese, respectively; and 23.4% and 15.8% had the presence of anxiety or depression symptoms, respectively (Table 1).

### Distribution of FokI polymorphism

**Figure 2:**
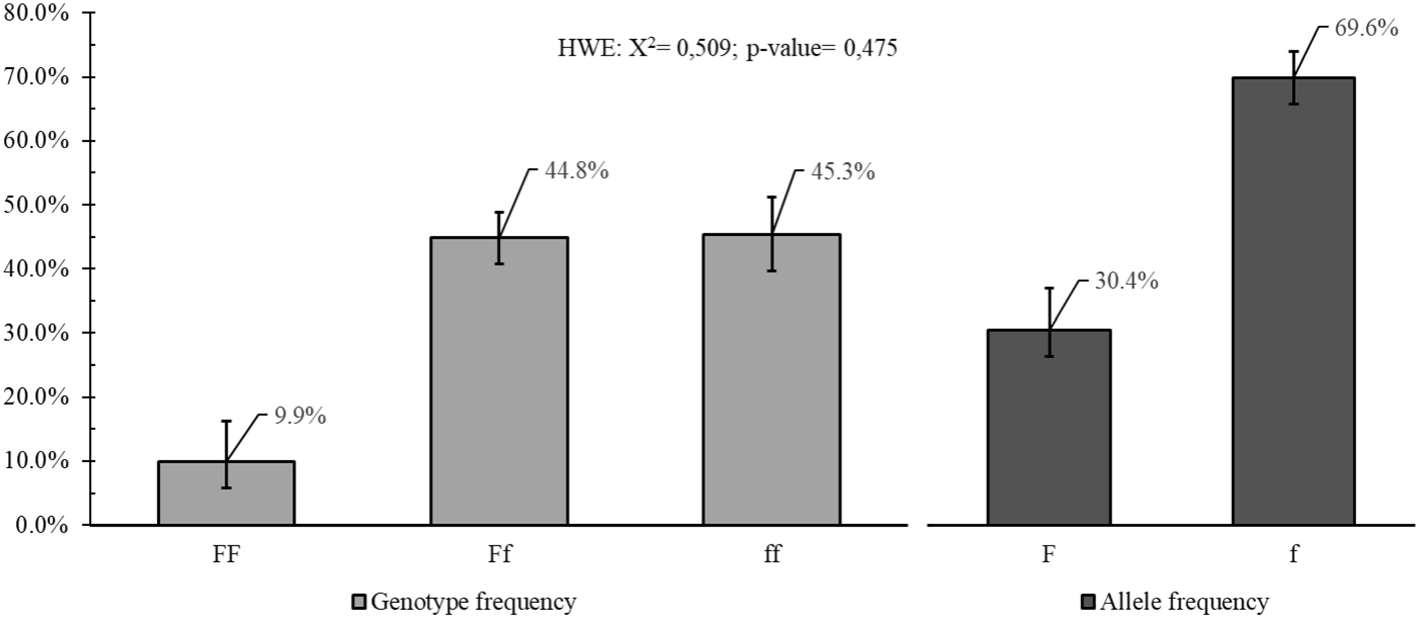
Distribution of *VDR* gene FokI polymorphism (rs2228570) genotype and allelic variants of the study population. Legend: HWE, Hardy–Weinberg equilibrium. Distributions of genotypes for FokI polymorphism did not deviate from expectations predicted by the HWE (p > 0.05) as determined by a chi-square test in both groups (X^2^: 0,509; p-value: 0,475).

The *VDR* gene FokI polymorphism (rs2228570) genotype frequency was 9.9% (5.8–16.3) for wild homozygote (AA, also called FF), 44.6% (41.0–49.1) for heterozygote (AG also called Ff), and 45.5% (39.3–51.0) for mutated homozygote (GG also called ff) (Fig. 2). The distribution of genotypes for the FokI polymorphism did not deviate from the expectations predicted by HWE (p > 0.05), as determined using a chi-square test in both groups (X^2^:0,509; p-value: 0,475) (Fig. 2).

### Association of vitamin D and FokI polymorphism with sleep quality

Considering the vitamin D deficiency and FokI polymorphism as explanatory variables, individuals with vitamin D deficiency had a 1.55 chance of poor sleep quality (OR_adjusted_: 1.55; 95% CI: 1.01–2.40), and individuals with the ff genotype had a 1.49 chance of poor sleep quality (Or_adjusted_: 1.49; 95% CI: 1.04–2.15) compared to their counterparts (vitamin D sufficiency and FF or Ff genotype). In a combined analysis, individuals with vitamin D deficiency and ff genotype had a higher chance of poor sleep quality when compared to the isolated factors than individuals with sufficient vitamin D and Ff or FF genotype (OR_adjusted_: 2.19; 95% CI: 1.27–3.76) (Table 2).

**Table 2:** Association between sleep quality with vitamin D levels and VDR polymorphism FokI (rs2228570) genotype.

	PSQI, mean (95%CI)*	Poor sleep quality			*p*
		Unadjusted	*p*	Adjusted**	
Vitamin D		**OR (95%CI)**		**OR (95%CI)**	**-**
Sufficiency	6.09 (5.92–6.52)	1.00		1.00	
Deficiency	7.02 (6.17–7.89)	1.21 (0.70–2.11)	0.489	1.55 (1.01–2.40)	**0.046**
FokI genotype		OR (95%CI)		OR (95%CI)	**-**
Ff or FF	6.34 (5.89–6.80)	1.00		1.00	
ff	6.43 (6.07–6.79)	1.16 (0.87–1.54)	0.310	1.49 (1.04–2.15)	**0.032**
Vitamin D and FokI genotype		OR (95%CI)	*p*	OR (95%CI)	*p*
Vitamin D sufficiency + Ff or FF	6.06 (5.84–6.57)	1.00		1.00	
Vitamin D sufficiency + ff	6.13 (5.80–6.69)	0.97 (0.50–1.90)	0.941	1.47 (0.82–2.61)	0.192
Vitamin D deficiency + Ff or FF	6.98 (5.53–8.34)	0.95 (0.62–1.46)	0.818	1.28 (0.78–2.14)	0.339
Vitamin D deficiency + ff	7.05 (6.33–7.93)	1.61 (1.12–2.34)	**0.012**	2.19 (1.27–3.76)	**0.005**

The outcome variable evaluated was poor sleep quality (PSQI > 5).

Collinearity among variables in the adjusted model evaluated by variance inflation factor (VIF) with the maximum remaining VIF = 1.56.

Bold values indicates p < 0.05 in logistic regression.

*PSQI* Pittsburgh sleep quality index, *OR* odds ratio, *CI* Confidence interval.

*Values are presented as the mean of PSQI score and 95% confidence interval.

**The directed acyclic graph (DAG) was used to support the theoretical model for the adjusted analysis between FokI polymorphism (explanatory variable) and poor sleep quality (outcome). Adjusted analysis by the following minimum and sufficient set of variables: age, sex, skin color, alcohol and tobacco consumption, body mass index, physical activity, sunlight and mental health.

**Figure 3:**
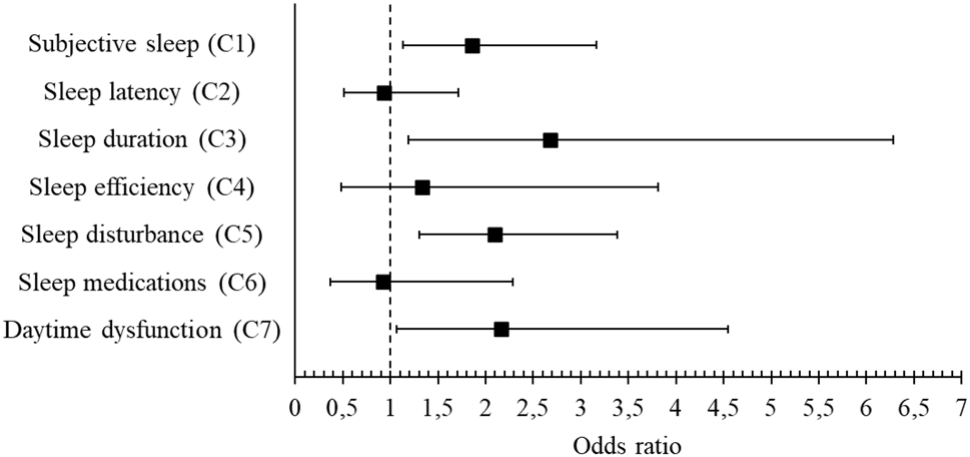
Association of vitamin D deficiency and ff genotype FokI polymorphism with moderate to difficulties Pittsburgh Sleep Quality Index subdomains. Performed logistic regression adjusted by age, sex, skin color, alcohol and tobacco consumption, body mass index, physical activity, sunlight and mental health. The outcome variable was moderate to difficulty in the sleep domain, explanatory variable the individuals with vitamin D deficiency and ff genotype FokI polymorphism, and the reference group was vitamin D sufficiency and FF or Ff genotype FokI polymorphism. The score for each domain ranges from 0 to 3 (no difficulty to severe difficulty), and a domain score ≥ 2 indicates moderate to difficulty in the sleep domain.

Furthermore, Fig. 3 shows the association of vitamin D and FokI polymorphisms with the moderate-to-difficult sleep subdomains of the PSQI. Therefore, we found that the combination of vitamin D deficiency and ff genotype was associated with poor subjective sleep quality (OR: 1.86; 95% CI: 1.07–3.16), shorter sleep duration (OR: 2.68; 95% CI: 1.19–6.27), greater sleep disturbances (OR: 2.10; 95% CI: 1.30–3.38), and more daytime dysfunction (OR: 2.15; 95% CI: 1.07–4.55).

## Discussion

To the best of our knowledge, this is the first population-based study to evaluate the association between vitamin D and *VDR* gene FokI polymorphism (rs2228570) and poor sleep quality in adults. We verified that vitamin D deficiency and the ff genotype of the FokI polymorphism were associated with poor sleep quality, and the combined analysis of the two factors increased the risk of poor sleep quality compared with the separate groups.

There is evidence that sleep duration is shorter in adults, older individuals, and school-aged children^5,18,24–30^ when vitamin D levels are low. As demonstrated by Gao et al. (2018), in a systematic review and meta-analysis of 9397 participants, individuals with vitamin D deficiency had a significantly increased chance for sleep disturbances [OR: 1.50; (95% CI: 1.31–1.72)], poor sleep quality OR: 1.59; [(95% CI: 1.23–2.05)], short sleep duration OR: 1.74; (95% CI: 1.30–2.32)], and excessive sleepiness [OR: 1.36; (95% CI: 1.12–1.65)]^2^. These results are supported by other studies conducted in different populations^5,18,24,25,30^. This evidence comes from different methods of sleep assessment, such as objective measurement^31^, self-reporting^32^, and systematic and meta-analytic analyses^2,33–35^.

Although several studies have demonstrated this association, the main predictive factors for this relationship have not yet been fully identified. One of the proposed hypotheses suggests a possible link between the VDR and areas of the brain responsible for regulating sleep, such as the hypothalamus, as the VDR has already been identified in these regions^36^. Furthermore, Fang et al. (2020) demonstrated that melatonin can bind directly to the VDR, mainly in the ligand-binding domain located at the C-terminus of the VDR, thus influencing its transcriptional activity^37^. Consequently, VDR may serve as a nuclear target for melatonin, with the FokI polymorphism potentially altering the affinity and conformation of VDR, thereby affecting its interaction with melatonin and the subsequent effects on sleep regulation.

Therefore, the VDR modulates the biological actions of vitamin D in various tissues and organs^38,39^, including during sleep. In this regard, genetic alterations of the VDR, such as the FokI polymorphism, are objects of interest because they can modify the pathway of action of vitamin D, altering its interaction with target tissues^40,41^, and potentially being one of the possible causes of the relationship between vitamin D and sleep quality and sleep disorders^12,42^.

The FokI polymorphism is located in exon 2, the first translation initiation codon, and, to date, is the only variant identified to alter the protein structure of the VDR^43^. It is also considered an independent molecular marker because it does not appear to be in linkage disequilibrium with any other *VDR* gene polymorphisms^44^. When its polymorphic form is present, there is a change in the start codon position, indicating that the VDR protein expression is three amino acids longer than that of the wild-type allele. To fate, most studies have demonstrated that the shorter version of the protein (wild-type allele A or F, depending on the terminology used), containing 424 amino acids, is more active, in terms of its activity as a transcriptional factor^45^, than the long form (polymorphism-type allele G or f), which contains 427 amino acids. Jurutka et al.^46^ demonstrated that a VDR protein with 424 amino acids interacts more efficiently with transcription factors, resulting in a more active VDR protein^46^.

The influence of FokI polymorphisms on protein function and signaling remains poorly understood. Recent studies have demonstrated that the FokI polymorphism may be associated with a wide variety of pathological and physiological phenotypes in different populations, such as variations in 1,25(OH)2D3 concentration^47^, variations in bone mineral density and fractures ^48^, alterations in insulin secretion and sensitivity to action^49^, metabolic syndrome^50^, and increased risk of some cancers^51^, depression, schizophrenia, bipolar disorder, and seasonal affective disorder.

In the present study, the polymorphic ff genotype of VDR FokI was associated with poor sleep quality. Most sleep disorders result from complex interactions between environmental and social factors and individual genetic predispositions^52^. Regarding vitamin D-related polymorphisms, there are yet to be studies evaluating the relationship between FokI polymorphisms and sleep quality. Only two small studies have investigated the role of *VDR* polymorphisms in other sleep disorders. In the first study, Kirac et al.^53^ investigated two *VDR* gene polymorphisms (FokI and BsmI) in 50 individuals with obstructive sleep apnea (OSA) and 50 healthy individuals. They showed that VDR polymorphisms were significantly associated with OSA^53^. A second study conducted by Abbas et al. (2019) recruited 240 Egyptian adults with OSA and 120 matched controls and found a gradient dose–response, as one mutated allele (Ff) increased the risk of OSA by more than two times (OR: 2.66, p = 0.03), and when both mutated alleles were present (ff) the risk of OSA was 10 times higher (OR: 10.59, p < 0.001)^12^.

The role of vitamin D in the synthesis of two neurotransmitters, serotonin and melatonin, may explain this association. Serotonin also regulates mood, anxiety, and cognition. Melatonin controls the circadian rhythm of sleep-wakefulness. These neurotransmitters are derived from the amino acid tryptophan. The enzyme tryptophan hydroxylase 2, which converts tryptophan into serotonin, is activated by vitamin D. Furthermore, vitamin D suppresses the enzyme arylalcylamine N-acetyltransferase, which converts serotonin into melatonin^54^. This mechanism may be influenced by the FokI polymorphism in the VDR gene. The mutated allele of this gene may decrease the sensitivity to vitamin D and its effects on serotonin and melatonin synthesis. This can result in serotoninergic overactivity during the day and melatoninergic hypoactivity at night^5^. These changes can impair mood and sleep, increasing the risk of psychiatric disorders and sleep disturbance^55^. However, as shown in our study, vitamin D deficiency may worsen this condition as it decreases the availability of vitamin D to bind to the VDR and regulate serotonin and melatonin synthesis. In addition, the FokI polymorphism may upregulate the expression of vitamin D target genes in cells by modulating processes, such as cell differentiation, inflammation, and the circadian rhythm^56^. For example, body clock genes that control sleep–wake cycles can be regulated by vitamin D. Thus, individuals with the FF or Ff genotypes are more sensitive to the effects of vitamin D than those with the ff genotype.

Therefore, the combination of vitamin D deficiency and the FokI polymorphism may be associated with sleep, especially in individuals with a genetic or environmental predisposition to this condition. However, considering the limited number of studies that have evaluated the influence of the FokI polymorphism on sleep, we suggest that the ff genotype should be the focus of further studies to better understand its influence on poor sleep quality, confirm this hypothesis, and elucidate the causal mechanisms involved. Therefore, considering that more than half of the evaluated population had the ff genotype for the FokI polymorphism, vitamin D levels must be assessed and maintained at sufficient values to avoid compromising sleep quality. Furthermore, prevention and treatment strategies for sleep disorders that take into account the interactions between vitamin D and serotoninergic and melatoninergic neurotransmitters must be developed. These strategies include vitamin D supplementation, adequate sunlight or artificial light exposure, cognitive behavioral therapy, and case-specific pharmacological therapy. Thus, we aimed to improve the quality of life of people affected by these conditions.

Our study has several strengths. The sample design was robust to the following: (i) a representative random sample of the resident population from different socioeconomic strata; (ii) assessment by household survey; (iii) face-to-face study in a COVID-19 pandemic scenario; (iv) large sample size of genetic marker research; and (v) Use of DAG to guide analysis plans. Furthermore, it is essential to understand genetic variations in mixed populations (populations with two or more different ethnic origins) because these populations may inherit genetic factors that increase their susceptibility or resistance to chronic diseases, such as cancer, cardiovascular disease, diabetes, autoimmune diseases, and psychiatric disorders^57^. However, although our findings provide relevant insights, this study has limitations in some areas that deserve attention. First, the sleep quality information obtained was self-reported; therefore, an individual’s perception may be overestimated or underestimated compared to objective measures. However, the evaluation of sleep quality needs to be performed subjectively because it considers factors intrinsic to individuals’ perception of their sleep^58–60^. Furthermore, although we adjusted the models for potential confounders using the counterfactual approach of the DAG, residual confounding from the design method could not be excluded. Another limitation is the assessment of only the FokI polymorphism of the VDR gene, which may not fully capture the genetic variability of VDR and its implications in sleep regulation and vitamin D responses. Future investigations should consider analyzing other VDR gene polymorphisms, such as BsmI, ApaI, and TaqI, which may also contribute to these processes. However, our study is the first to establish a correlation between the VDR FokI polymorphism and sleep quality in a representative sample of the Brazilian adult population using a robust methodology (probabilistic sampling from a population-based household survey). This is significant because many existing studies are based on small, selected, or clinical samples, which can introduce selection bias and confounding factors.

## Conclusion

This study suggests that the ff genotype of the *VDR* FokI (rs2228570) polymorphism is associated with poor sleep quality in adults. Furthermore, this genotype is associated with vitamin D deficiency, thereby increasing the risk of poor sleep. Therefore, considering that more than half of the evaluated population had the ff genotype of the FokI polymorphism, vitamin D levels must be assessed and maintained at sufficient levels to avoid sleep quality-related impairments.

### Supplementary Information

Supplementary Table S1.

Supplementary Table S2.

Supplementary Table S3.

## Data Availability

The data supporting the conclusions of this study can be obtained from the corresponding author on reasonable request.
