# Supplementary material for: The role of interaction between vitamin D and VDR FokI gene polymorphism (rs2228570) in sleep quality of adults
Source: Sci Rep. 2024 Apr 7;14:8141. doi: 10.1038/s41598-024-58561-2 (PMC10999418; doi:10.1038/s41598-024-58561-2)
Supplement: Supplementary file 1 — Supplementary Table S1. [file 41598_2024_58561_MOESM1_ESM.docx]

| **Supplementary Table 1:** Health-related pathways overview illustrated in the directed acyclic graph (DAG) | | |
| --- | --- | --- |
| **Pathway** | **Rationale** | **References** |
| Age → Alcohol and tobacco consumption | Alcohol and tobacco consumption tends to increase with age due to factors such as social changes, stress and the availability of financial resources. In addition, prolonged consumption throughout life can result in greater dependence and excessive consumption. | [doi.org/10.1007/s00213-011-2236-1](http://doi.org/10.1007/s00213-011-2236-1); [doi.org/10.1186/s12889-023-17232-z](http://doi.org/10.1186/s12889-023-17232-z) |
| Age → Body mass index | BMI tends to increase with age due to changes in metabolism, loss of muscle mass, and hormonal changes associated with aging. | [doi.org/10.1146/annurev.nutr.22.010402.102715](http://doi.org/10.1146/annurev.nutr.22.010402.102715); [doi.org/10.1093/ageing/afw042](http://doi.org/10.1093/ageing/afw042) |
| Age → Chronic disease | Many chronic diseases, such as cardiovascular disease, diabetes, and certain types of cancer, have an increased incidence with age due to the accumulation of risk factors over a lifetime and the deterioration of physiological systems. | [doi.org/10.1186/s12889-019-7762-5](http://doi.org/10.1186/s12889-019-7762-5) |
| Age → Exposure to sunlight | Exposure to sunlight may decrease with age due to factors such as reduced mobility, sun-protective behaviors or increased time spent indoors. | [doi.org/10.1038/s41598-020-70329-y](http://doi.org/10.1038/s41598-020-70329-y); [doi.org/10.1093/ajcn/80.6.1678S](http://doi.org/10.1093/ajcn/80.6.1678S); [doi.org/10.1111/j.1600-0781.2010.00518.x](http://doi.org/10.1111/j.1600-0781.2010.00518.x) |
| Age → Symptoms of anxiety or depression | Studies show that younger people are more likely to suffer from mental disorders. In general, cases of depression and anxiety begin in adolescence and young adulthood (18-25 years) and may be related to hormonal, social, or academic factors. This is also the age of graduation, a time when young people face a number of life changes. | [doi.org/10.1097/YCO.0b013e32816ebc8c](http://doi.org/10.1097/YCO.0b013e32816ebc8c); [doi.org/10.1038/s41380-021-01161-7](http://doi.org/10.1038/s41380-021-01161-7) |
| Age → Vitamin D | The skin's ability to synthesize vitamin D from sunlight decreases with age due to changes in skin structure and decreased sun exposure. In addition, older people may have a lower dietary intake of vitamin D. | [doi.org/10.1056/NEJMra070553](http://doi.org/10.1056/NEJMra070553); [doi.org/10.1007/s00198-009-0954-6](http://doi.org/10.1007/s00198-009-0954-6) |
| Alcohol and tobacco consumption → Chronic diseases | Alcohol and tobacco consumption are established risk factors for a variety of chronic diseases, including cardiovascular diseases, chronic respiratory diseases and certain types of cancer. Both behaviors are associated with inflammatory processes and oxidative damage that contribute to the development of these health conditions. | [doi.org/10.3390/nu14091954](http://doi.org/10.3390/nu14091954); [doi.org/10.5888/pcd19.220086](http://doi.org/10.5888/pcd19.220086) |
| Alcohol and tobacco consumption → Sleep quality | Excessive alcohol consumption and smoking are associated with sleep disorders such as insomnia and sleep fragmentation. Alcohol can interfere with sleep patterns and induce nocturnal awakenings, while nicotine can have stimulating effects that impair sleep quality. | [doi.org/10.1017/S1368980020004553](http://doi.org/10.1017/S1368980020004553); [doi.org/10.1016/j.sleepe.2022.100028](http://doi.org/10.1016/j.sleepe.2022.100028); [doi.org/10.7759/cureus.49162](http://doi.org/10.7759/cureus.49162); [doi.org/10.5888/pcd19.220086](http://doi.org/10.5888/pcd19.220086) |
| Body mass index → Chronic diseases | Obesity is an important risk factor for a variety of chronic diseases, including type 2 diabetes, cardiovascular diseases and certain types of cancer. Being overweight is associated with chronic inflammatory processes and metabolic dysfunction, which contribute to the development of these health conditions. | [doi.org/10.1186/s12916-021-02188-x](http://doi.org/10.1186/s12916-021-02188-x); [doi.org/10.1016/j.jmwh.2010.05.001](http://doi.org/10.1016/j.jmwh.2010.05.001) |
| Body mass index → Vitamin D | Obesity is associated with reduced vitamin D levels due to the sequestration of vitamin D in adipose tissue. In addition, individuals with a high BMI may have less exposure to sunlight due to reduced mobility or sun protection behaviors, resulting in lower cutaneous vitamin D synthesis. As well as decreased vitamin D synthesis in adipose tissue and the liver. | [doi.org/10.1001/jamanetworkopen.2022.50681](http://doi.org/10.1001/jamanetworkopen.2022.50681); [doi.org/10.1111/obr.12239](http://doi.org/10.1111/obr.12239); [doi.org/10.1007/s13679-021-00433-1](http://doi.org/10.1007/s13679-021-00433-1) |
| Chronic diseases → Vitamin D | Chronic illnesses can lead to vitamin D deficiency in various ways. For example, conditions that affect the absorption of nutrients, such as inflammatory bowel diseases, can reduce the body's ability to absorb vitamin D from food. In addition, some chronic diseases can limit exposure to the sun, which is crucial for vitamin D synthesis in the skin. The chronic inflammation associated with these diseases can interfere with the metabolization of vitamin D, as well as increasing the body's need for vitamin D, due to its role in modulating the immune response and reducing inflammation. | [doi.org/10.1007/s00223-021-00844-1](http://doi.org/10.1007/s00223-021-00844-1); [doi.org/10.1007/s13197-017-2840-0](http://doi.org/10.1007/s13197-017-2840-0); [doi.org/10.1007/s40257-017-0323-8](http://doi.org/10.1007/s40257-017-0323-8) |
| Exposure to sunlight → Sleep quality | Exposure to sunlight during the day helps regulate circadian rhythms and promotes the production of melatonin at night, which is essential for healthy, quality sleep. | [doi.org/10.1002/jbio.201900102;](http://doi.org/10.1002/jbio.201900102;) [doi.org/10.1016/j.sleep.2006.11.017](http://doi.org/10.1016/j.sleep.2006.11.017); [doi.org/10.1007/s11818-019-00215-x](http://doi.org/10.1007/s11818-019-00215-x) |
| Exposure to sunlight → Symptoms of anxiety or depression | Sunlight is an important source of vitamin D, which plays a role in mood regulation and brain function. Additionally, sunlight regulates the circadian rhythm, which is crucial for healthy sleep and mood balance. Biological mechanisms suggest that sunlight affects the production of neurotransmitters like serotonin, which is associated with well-being and happiness. Lack of sunlight exposure, especially during winter months, can lead to Seasonal Affective Disorder (SAD), a form of depression related to seasonal changes. Furthermore, sunlight exposure can have psychological effects, such as increasing social contact and physical activity, both beneficial for mental health. | [doi.org/10.2147/RMHP.S420018](http://doi.org/10.2147/RMHP.S420018); [doi.org/10.1038/s44220-023-00135-8](http://doi.org/10.1038/s44220-023-00135-8); [doi.org/10.3390/psychiatryint3010008](http://doi.org/10.3390/psychiatryint3010008) |
| Exposure to sunlight → Vitamin D | Vitamin D synthesis in the skin is triggered by exposure to ultraviolet (UV) radiation from the sun. Adequate sun exposure is the main source of vitamin D for most people. | [doi.org/10.4161/derm.24494](http://doi.org/10.4161/derm.24494); [doi.org/10.1046/j.1525-1497.2002.20731.x](http://doi.org/10.1046/j.1525-1497.2002.20731.x); [doi.org/10.1016/j.jsbmb.2010.04.002](http://doi.org/10.1016/j.jsbmb.2010.04.002) |
| FokI polymorphism → Sleep quality | Although the direct relationship between the FokI polymorphism and sleep quality is not well established, it is known that variations in the vitamin D receptor gene can influence metabolic and immunological processes, which in turn can affect sleep. It can also affect sensitivity to vitamin D, which in turn can modulate sleep quality through its effects on the central nervous system. | [doi.org/10.1002/eji.200636043](http://doi.org/10.1002/eji.200636043); [doi.org/10.1089/omi.2018.0184](http://doi.org/10.1089/omi.2018.0184); [doi.org/10.4103/ejcdt.ejcdt_152_18](http://doi.org/10.4103/ejcdt.ejcdt_152_18) |
| Genetic inheritance → Body mass index | Body mass index (BMI) is strongly influenced by genetics, with studies identifying genomic locations associated with BMI in childhood and adulthood. These locations can affect energy metabolism and fat storage, increasing an individual's predisposition to weight gain and obesity | [doi.org/10.1371/journal.pmed.1002215](http://www.doi.org/10.1371/journal.pmed.1002215); [doi.org/10.1093/hmg/ddv472](http://www.doi.org/10.1093/hmg/ddv472) |
| Genetic inheritance → Chronic diseases | Many chronic diseases have a genetic basis, including type 2 diabetes, cardiovascular diseases and certain types of cancer. Genetic variants can increase the risk of developing these diseases throughout life. In addition, epigenetic changes can have transgenerational effects, affecting susceptibility to chronic diseases in future generations. | [doi.org/10.1007/978-1-84882-644-1_1](http://doi.org/10.1007/978-1-84882-644-1_1); [doi.org/10.1093/gerona/glx154](http://doi.org/10.1093/gerona/glx154); [doi.org/10.1038/s41591-024-02796-z](http://doi.org/10.1038/s41591-024-02796-z) |
| Genetic inheritance → FokI polymorphism | Polymorphism is a genetic variation inherited from parents to offspring. Following Mendel's laws, each parent contributes one allele to their child's genotype. If one or both parents have the allele with the FokI polymorphism, there is a possibility that this genetic trait will be passed on to their offspring. | [doi.org/10.1007/978-1-4684-0814-0_8](http://doi.org/10.1007/978-1-4684-0814-0_8); [doi.org/10.1016/B978-0-12-374984-0.01189-X](http://doi.org/10.1016/B978-0-12-374984-0.01189-X) |
| Genetic inheritance → Skin color | Skin pigmentation is a complex trait influenced by multiple genes and environmental exposure to ultraviolet radiation. Genetic variations at specific loci can lead to differences in skin pigmentation, adapting to different levels of sun exposure throughout human evolution. | [doi.org/10.1186/s41065-017-0036-2](http://doi.org/10.1186/s41065-017-0036-2); [doi.org/10.1007/s00439-017-1808-5](http://doi.org/10.1007/s00439-017-1808-5) |
| Sex → Body mass index | Gender differences can influence BMI due to biological, behavioral and social factors. For example, hormonal differences between men and women can affect the distribution of body fat. In addition, cultural norms and social expectations regarding body weight can vary between the sexes. | [doi.org/10.1155/2019/1360328](http://doi.org/10.1155/2019/1360328); [doi.org/10.1186/s12889-019-7351-7](http://doi.org/10.1186/s12889-019-7351-7); [doi.org/10.3945/an.112.002063](http://doi.org/10.3945/an.112.002063) |
| Sex → Sleep quality | Gender can influence sleep quality due to biological, hormonal and behavioral differences between men and women. For example, hormonal fluctuations throughout the menstrual cycle can affect women's sleep patterns. In addition, social factors, such as family responsibilities and gender roles, can also influence sleep patterns. | [doi.org/10.3390/life12122003](http://doi.org/10.3390/life12122003); [doi.org/10.1007/978-3-030-40842-8_5](http://doi.org/10.1007/978-3-030-40842-8_5) |
| Sex → Symptoms of anxiety or depression | The female sex is associated with higher levels of mental disorders when compared to the male sex. This difference can be explained by genetic and biological factors, including hormonal characteristics, personal stigma, greater sensitivity to traumatic events, as well as the fact that women recognize mental health problems better and seek more help from health services compared to men. | [doi.org/10.1093/pubmed/fdab406](http://doi.org/10.1093/pubmed/fdab406); [doi.org/10.1017/CBO9780511984945.017](http://doi.org/10.1017/CBO9780511984945.017); [doi.org/10.1016/j.bpsgos.2023.100283](http://doi.org/10.1016/j.bpsgos.2023.100283) |
| Sex → Vitamin D | Gender differences can influence vitamin D levels due to differences in body composition, sun exposure, health behaviors and hormonal factors. Studies have shown that women tend to have lower serum vitamin D levels compared to men, possibly due to differences in body fat distribution and more frequent use of sunscreen. | [doi.org/10.3390/nu11123034](http://doi.org/10.3390/nu11123034); [doi.org/10.1017/S0007114522000149](http://doi.org/10.1017/S0007114522000149); [doi.org/10.1159/000458765](http://doi.org/10.1159/000458765) |
| Skin color → FokI polymorphism | Skin color is often used as an indirect indicator of genetic inheritance, especially in population-based association studies. Genetic variations affecting skin color may be linked to other genetic polymorphisms, such as FokI. Studies show that the frequency of certain polymorphisms, including FokI, varies significantly between different ethnic groups, which suggests that skin color may reflect underlying genetic inheritance that also influences the presence of polymorphisms such as FokI. These differences in polymorphism frequencies are observed in global genetic variation data, such as PubMed's SNP Consortium Map | [doi.org/10.1038/nature15393](http://doi.org/10.1038/nature15393); [doi.org/10.1038/nature04226](http://doi.org/10.1038/nature04226) |
| Skin color → Sleep quality | Skin color can influence sleep quality for various reasons. Ethnic/racial minorities often face discrimination, which can lead to chronic stress and anxiety, negatively affecting sleep. Socioeconomically, individuals with darker skin can face higher levels of stress, which can result in sleep disturbances. In addition, factors such as unequal access to healthcare and more stressful living environments can also contribute to poorer sleep quality. | [doi.org/10.1093/oso/9780190930448.001.0001](http://doi.org/10.1093/oso/9780190930448.001.0001); [doi.org/10.1186/1471-2458-10-475](http://doi.org/10.1186/1471-2458-10-475) ; [doi.org/10.1007/s11469-020-00378-x](http://doi.org/10.1007/s11469-020-00378-x) |
| Skin color → Symptoms of anxiety or depression | Non-white skin color is related to a higher prevalence of mental disorders such as anxiety and depression. This association can be explained, at least in part, by the perceived racial discrimination that racial/ethnic minorities suffer compared to other groups. Racial/ethnic minorities experience crime, hate speech and microaggressions more often, which can negatively affect mental well-being. | [doi.org/10.1016/j.socscimed.2022.115387](http://doi.org/10.1016/j.socscimed.2022.115387); [doi.org/10.1086/682162](http://doi.org/10.1086/682162); [doi.org/10.1590/1413-812320172212.19782016](http://doi.org/10.1590/1413-812320172212.19782016) |
| Skin color → Vitamin D | Skin pigmentation influences the amount of vitamin D produced in response to exposure to sunlight. Individuals with darker skin have a higher melanin content, which can block the synthesis of vitamin D in the skin. This can lead to lower levels of vitamin D in people with darker skin, especially in areas with less sun exposure. | [doi.org/10.1590/0004-2730000003320](http://doi.org/10.1590/0004-2730000003320); [doi.org/10.1159/000354750](http://doi.org/10.1159/000354750) |
| Symptoms of anxiety or depression → Sleep quality | Anxiety disorders and depression are often associated with sleep disorders, such as insomnia and non-restorative sleep. Anxiety and excessive rumination can interfere with the ability to fall asleep and maintain deep sleep, while depression can lead to irregular sleep patterns and early awakenings. | [doi.org/10.31887/DCNS.2008.10.3/dnutt](http://doi.org/10.31887/DCNS.2008.10.3/dnutt); [doi.org/10.1017/S2045796021000810](http://doi.org/10.1017/S2045796021000810); |
| Vitamin D → Sleep quality | Vitamin D deficiency has been associated with sleep disorders such as insomnia and poor sleep quality. Vitamin D plays an important role in regulating circadian rhythms, modulating pain and inflammation, and in the production of melatonin which can affect sleep. In addition, vitamin D receptors are present in areas of the brain involved in sleep regulation. | [doi.org/10.3390/nu14051076](http://doi.org/10.3390/nu14051076); [doi.org/10.3390/nu10101395](http://doi.org/10.3390/nu10101395); [doi.org/10.1530/JOE-16-0514](http://doi.org/10.1530/JOE-16-0514) |
| Vitamin D supplementation → Vitamin D | This is a trivial relationship, where vitamin D supplementation increases blood levels of this vitamin. Studies have shown that supplementation is effective in correcting vitamin D deficiencies. | [doi.org/10.1056/NEJMra070553](http://doi.org/10.1056/NEJMra070553); [doi.org/10.1016/j.jsbmb.2017.01.021](http://doi.org/10.1016/j.jsbmb.2017.01.021) |
