# Supplementary material for: The role of interaction between vitamin D and VDR FokI gene polymorphism (rs2228570) in sleep quality of adults
Source: Sci Rep. 2024 Apr 7;14:8141. doi: 10.1038/s41598-024-58561-2 (PMC10999418; doi:10.1038/s41598-024-58561-2)
Supplement: Supplementary file 2 — Supplementary Table S2. [file 41598_2024_58561_MOESM2_ESM.docx]

| **Supplementary Table 2.** Sociodemographic and health conditions according vitamin D levels in adults during the COVID-19 pandemic, COVID-Inconfidentes Study (2020). | | | |
| --- | --- | --- | --- |
| **Characteristics** | **Vitamin D** | | **p-value*** |
|  | **Sufficiency**  **% (95%CI)** | **Deficiency**  **% (95%CI)** |  |
| **Total** | 80.2 (75.1-84.5) | 19.8 (15.5-24.9) | - |
| Sociodemographic |  |  |  |
| **Sex** |  |  |  |
| Male | 47.9 (39.3-56.6) | 47.3 (42.3-62.8) | 0.933 |
| Female | 52.1 (43.4-60.7) | 52.7 (42.3-62.8) |  |
| **Age** |  |  |  |
| Years, mean (95%CI) | 40.2 (38.8-41.6) | 52.4 (49.6-55.2) | **< 0.001** |
| 18 to 34 years | 35.0 (30.0-40.2) | 22.3 (14.2-33.3) | **0.001** |
| 35 to 59 years | 46.5 (41.4-51.7) | 35.6 (28.1-44.0) |  |
| ≥ 60 years | 18.5 (14.8-23.0) | 42.1 (35.0-49.4) |  |
| **Skin color ^a^** |  |  |  |
| White | 23.9 (18.7-30.0) | 33.6 (22.2-47.3) | 0.163 |
| Black | 20.2 (14.7-27.1) | 20.6 (15.1-27.5) |  |
| Brown | 49.5 (41.7-57.4) | 43.2 (32.6-54.5) |  |
| Others | 6.4 (4.4-9.1) | 2.6 (1.4-4.4) |  |
| **Marital status ^b^** |  |  |  |
| Married  Not married | 53.0 (45.8-60.0) | 56.5 (44.4-67.9) | 0.604 |
|  | 47.0 (40.0-54.2) | 43.5 (32.1-55.6) |  |
| **Education** |  |  |  |
| 0 to 8 years | 29.7 (24.8-35.2) | 33.6 (26.0-42.1) | **0.003** |
| 9 to 11 years | 40.3 (36.2-44.4) | 37.6 (26.6-50.0) |  |
| > 12 years | 30.0 (24.1-36.7) | 28.9 (20.5-38.9) |  |
| **Family Income ^c^** |  |  |  |
| ≤ 2 MW | 45.6 (40.1-51.1) | 43.0 (32.9-53.6) | 0.867 |
| > 2 to ≤ 4 MW | 28.0 (22.2-34.7) | 34.9 (26.7-44.1) |  |
| > 4 MW | 26.4 (20.9-32.8) | 22.1 (16.4-29.2) |  |
| Health conditions |  |  |  |
| **FokI polymorphism^d^** |  |  |  |
| FF or Ff | 54.8 (48.0-61.5) | 52.7 (42.2-63.0) | 0.725 |
| ff | 45.2 (38.5-52.0) | 47.3 (37.0-57.8) |  |
| **Sleep quality ^e^** |  |  |  |
| Good | 47.6 (43.5-51.6) | 43.6 (31.6-56.5) | 0.563 |
| Poor | 52.4 (48.4-56.5) | 56.4 (43.5-68.4) |  |
| **Chronic diseases ^f^** |  |  |  |
| No | 51.4 (43.7-59.1) | 33.2 (25.2-42.3) | **0.004** |
| Yes | 48.6 (40.9-56.3) | 66.8 (57.7-74.8) |  |
| **Smoking** |  |  |  |
| No | 84.3 (79.7-88.9) | 76.7 (68.6-86.0) | 0.227 |
| Yes | 15.6 (11.1-20.3) | 23.3 (14.0-31.4) |  |
| **Alcohol consumption** |  |  |  |
| No | 39.2 (29.5-43.9) | 51.8 (42.6-60.9) | **0.012** |
| Yes | 60.8 (56.1-70.5) | 48.2 (39.1-57.4) |  |
| **Body mass index (BMI) ^g^** |  |  |  |
| BMI, kg/m² | 26.2 (25.7-26.7) | 27.6 (26.6-28.5) | **0.018** |
| Eutrophic | 43.0 (34.1-52.5) | 39.9 (30.4-50.3) | **0.023** |
| Underweight | 1.7 (1.1-2.6) | 4.2 (2.5-6.8) |  |
| Overweight | 40.2 (29.7-51.7) | 28.5 (22.4-35.4) |  |
| Obesity | 15.1 (11.6-19.5) | 27.4 (20.1-36.1) |  |
| **Mental health ^h^** |  |  |  |
| Presence of anxiety symptoms | 18.9 (14.0-25.0) | 31.6 (25.3-38.6) | **0.005** |
| Presence of depression symptoms | 12.4 (9.0-16.9) | 22.0 (15.7-29.9) | **0.024** |
| **Exposure to sunlight ^i^** |  |  |  |
| Daily sunlight (hours/day) | 1.8 (1.1-2.4) | 1.7 (0.9-2.4) | 0.963 |
| **Vitamin D supplementation** |  |  |  |
| No | 92.5 (90.0-94.4) | 97.8 (95.9-98.8) | **< 0.001** |
| Yes | 7.5 (5.6-10.0) | 2.2 (1.2-4.1) |  |
| Vitamin D deficiency: 25(OH)D < 20 ng/mL for a healthy population and < 30 ng/mL for groups at risk for vitamin D deficiency.  * p-value of Pearson's chi-square test  # T-test was performed.  ^a^ The participants were categorized into those with white, black, brown, and others race/skin colors (indigenous and yellows).  ^b^ Not married: Widowed, divorced, single  ^c^ Minimum wage value: BRL 1,045.00 ≈ USD 194.25 (1 USD = 5.3797 BRL)  ^d^ Genotype frequency (FokI): FF or AA- homozygous wild, Ff or AG- heterozygous and ff or GG- homozygous mutant  ^e^ Poor sleep quality determined by PSQI ≥ 5.  ^f^ Chronic disease: medical diagnosis of at least one chronic disease  ^g^ Underweight (BMI < 18.5 kg/m² if < 60 years or BMI < 22.0 kg/m² if > 60 years), eutrophic (BMI 18.5-24.9 kg/m² if < 60 years or BMI 22.0-27.9 kg/m² if > 60 years), overweight (BMI 25.0-29.9 kg/m² if < 60 years or BMI 28.0-29.9 kg/m² if > 60 years), obese (BMI > 30.0 kg/m²).  ^h^ The GAD-7 and PHQ-9 scales, were used to determining the presence of anxiety and depression symptoms, respectively.  ^i^ Daily sunlight was calculated from the following formula: [weekly frequency of sunlight (0 to 7 days) x daily time of sunlight (minutes)/7]). | | | |
