# Supplementary material for: The role of interaction between vitamin D and VDR FokI gene polymorphism (rs2228570) in sleep quality of adults
Source: Sci Rep. 2024 Apr 7;14:8141. doi: 10.1038/s41598-024-58561-2 (PMC10999418; doi:10.1038/s41598-024-58561-2)
Supplement: Supplementary file 3 — Supplementary Table S3. [file 41598_2024_58561_MOESM3_ESM.docx]

| **Supplementary Table 3.** Sociodemographic and health conditions according FokI polymorphism (rs2228570 A>G) in adults during the COVID-19 pandemic, COVID-Inconfidentes Study (2020). | | | |
| --- | --- | --- | --- |
| **Characteristics** | **FokI polymorphism** | | **p-value*** |
|  | **Ff or FF**  **% (95%CI)** | **ff**  **% (95%CI)** |  |
| **Total** | 54.5 (48.4-60.5) | 45.5 (39.5-51.6) | - |
| Sociodemographic |  |  |  |
| **Sex** |  |  |  |
| Male | 49.8 (38.0-61.7) | 45.9 (40.5-51.5) | 0.549 |
| Female | 50.2 (38.3-62.0) | 54.1 (48.5-59.5) |  |
| **Age** |  |  |  |
| Years, mean (95%CI) | 43.8 (42.0-45.5) | 44.0 (42.0-45.9) | 0.864# |
| 18 to 34 years | 38.2 (31.9-44.8) | 32.5 (27.0-38.5) | 0.276 |
| 35 to 59 years | 45.0 (38.8-51.4) | 47.6 (42.0-53.2) |  |
| ≥ 60 years | 16.9 (12.7-22.1) | 19.9 (15.9-24.6) |  |
| **Skin color ^a^** |  |  |  |
| White | 30.0 (22.0-39.5) | 21.0 (16.3-26.6) | 0.053 |
| Black, brown and others | 70.0 (60.5-78.0) | 79.0 (73.4-83.7) |  |
| **Marital status ^b^** |  |  |  |
| Married  Not married | 54.4 (43.6-64.8) | 51.8 (45.3-58.2) | 0.685 |
|  | 45.6 (35.2-56.4) | 48.2 (41.8-54.7) |  |
| **Education** |  |  |  |
| 0 to 8 years | 28.4 (22.0-35.8) | 31.2 (24.9-38.2) | 0.451 |
| 9 to 11 years | 42.7 (35.7-50.0) | 36.7 (31.4-42.3) |  |
| > 12 years | 28.9 (21.4-37.7) | 32.1 (26.3-38.6) |  |
| **Family Income ^c^** |  |  |  |
| ≤ 2 MW | 42.6 (36.5-49.0) | 45.9 (38.8-53.1) | 0.747 |
| > 2 to ≤ 4 MW | 31.2 (25.4-40.0) | 30.3 (25.6-35.5) |  |
| > 4 MW | 26.2 (19.8-33.8) | 23.8 (18.8-29.8) |  |
| Health conditions |  |  |  |
| **Vitamin D ^d^** |  |  |  |
| Mean, ng/dL | 26.2 (24.8-27.6) | 26.0 (25.0-27.2) | 0.895 |
| Sufficiency (> 20 ng/dL) | 80.2 (72.6-86.9) | 79.2 (74.5-83.3) | 0.710 |
| Deficiency (< 20 ng/dL) | 19.3 (13.1-27.4) | 20.8 (16.7-25.5) |  |
| **Sleep quality ^e^** |  |  |  |
| Good | 47.9 (42.3-53.4) | 44.7 (39.1-50.5) | 0.401 |
| Poor | 52.1 (46.6-57.7) | 55.3 (49.5-60.9) |  |
| **Chronic diseases ^f^** |  |  |  |
| No | 65.8 (57.1-73.6) | 53.9 (47.8-59.8) | **0.029** |
| Yes | 34.2 (26.4-42.9) | 43.1 (40.2-52.2) |  |
| **Smoking** |  |  |  |
| No | 84.4 (77.0-89.7) | 80.7 (74.8-85.4) | 0.369 |
| Yes | 15.6 (10.3-22.9) | 19.3 (14.6-25.2) |  |
| **Alcohol consumption** |  |  |  |
| No | 41.0 (30.8-52.1) | 42.6 (37.7-47.6) | 0.798 |
| Yes | 58.9 (47.9-69.2) | 57.4 (52.4-62.3) |  |
| **Body mass index (BMI) ^g^** |  |  |  |
| BMI (kg/m²), | 26.7 (26.1-27.3) | 26.3 (25.8-26.8) | 0.373 |
| Eutrophic | 41.5 (30.6-53.3) | 45.1 (38.6-51.7) | 0.666 |
| Underweight | 2.3 (1.3-4.2) | 2.8 (1.8-4.2) |  |
| Overweight | 39.0 (26.2-53.5) | 33.3 (27.5-39.8) |  |
| Obesity | 17.3 (12.4-23.4) | 18.8 (15.0-23.4) |  |
| **Mental health ^h^** |  |  |  |
| Presence of anxiety symptoms | 23.9 (17.9-31.2) | 22.5 (17.8-27.7) | 0.723 |
| Presence of depression symptoms | 18.0 (12.5-25.2) | 13.9 (11.0-17.5) | 0.233 |
| **Exposure to sunlight ^i^** |  |  |  |
| Daily sunlight (hours/day) | 1.9 (1.0-2.9) | 1.4 (1.2-1.7) | 0.276 |
| **Vitamin D supplementation** |  |  |  |
| No | 93.6 (90.9-95.6) | 93.0 (90.2-95.1) | 0.685 |
| Yes | 6.4 (4.4-9.1) | 6.7 (4.9-9.8) |  |
| Genotype frequency (FokI): FF or AA- homozygous wild, Ff or AG- heterozygous and ff or GG- homozygous mutant  * p-value of Pearson's chi-square test  # T-test was performed.  ^a^ The participants were categorized into those with white, black, brown, and others race/skin colors (indigenous and yellows).  ^b^ Not married: Widowed, divorced, single  ^c^ Minimum wage value: BRL 1,045.00 ≈ USD 194.25 (1 USD = 5.3797 BRL)  ^d^ Vitamin D deficiency: 25(OH)D < 20 ng/mL for a healthy population and < 30 ng/mL for groups at risk for vitamin D deficiency.  ^f^ Poor sleep quality determined by PSQI ≥ 5.  ^g^ Chronic disease: medical diagnosis of at least one chronic disease  ^h^ Underweight (BMI < 18.5 kg/m² if < 60 years or BMI < 22.0 kg/m² if > 60 years), eutrophic (BMI 18.5-24.9 kg/m² if < 60 years or BMI 22.0-27.9 kg/m² if > 60 years), overweight (BMI 25.0-29.9 kg/m² if < 60 years or BMI 28.0-29.9 kg/m² if > 60 years), obese (BMI > 30.0 kg/m²).  ^i^ The GAD-7 and PHQ-9 scales, were used to determining the presence of anxiety and depression symptoms, respectively.  ^j^ Daily sunlight was calculated from the following formula: [weekly frequency of sunlight (0 to 7 days) x daily time of sunlight (minutes)/7]). | | | |
